# Supplementary material for: Identifying the critical state of cancers by single-sample Markov flow entropy
Source: PeerJ. 2023 Jul 24;11:e15695. doi: 10.7717/peerj.15695 (PMC10373650; doi:10.7717/peerj.15695)
Supplement: Supplemental Information 1 — [Step 1] The first step is to categorize sample s into a long-survival group and a short-survival group based on their clinical information, that is, the samples with a survival time greater than the median survival time were deemed as the long-survival group, and vice the samples with a survival time less than the median survival time were considered as the short-survival group. [Step 2] The second step is to find candidate biomarkers. For each sample, the signaling genes (top 5% genes with the highest sMFE value) that non-differently expressed were selected as a set of candidate genes. The samples were divided into two groups (identified samples and unidentified samples) according to whether their signaling genes contained the given candidate gene i, i.e., the signaling genes of identified samples included the given candidate gene i, while unidentified samples didn’t. The given candidate gene i is regarded as the candidate biomarker when it satisfies the following formula. n/(n+m) > A or m/(n+m) > A, where n denotes the number of identified samples from the long-survival group, and m denotes the number of identified samples from the short-survival group. The A is an empirical parameter and set as 0.6. [Step 3] The third step is to identify two types of biomarkers (optimistic sMFE biomarkers and pessimistic sMFE biomarkers) from candidate biomarkers. Specifically, for the candidate biomarker (gene j), the prognosis results respectively based on identified samples and unidentified samples were exhibited and compared through Kaplan–Meier (log-rank) survival analysis. If the survival curve of identified samples were significantly longer than that of unidentified samples (P-value <0.05), gene j was perceived as an optimistic sMFE biomarker (O-sMFE). Similarly, if the survival curve of identified samples was statistically shorter than that of unidentified samples (P-value < 0.05), gene j was perceived as a pessimistic sMFE biomarker (P-sMFE). [file peerj-11-15695-s001.pdf]

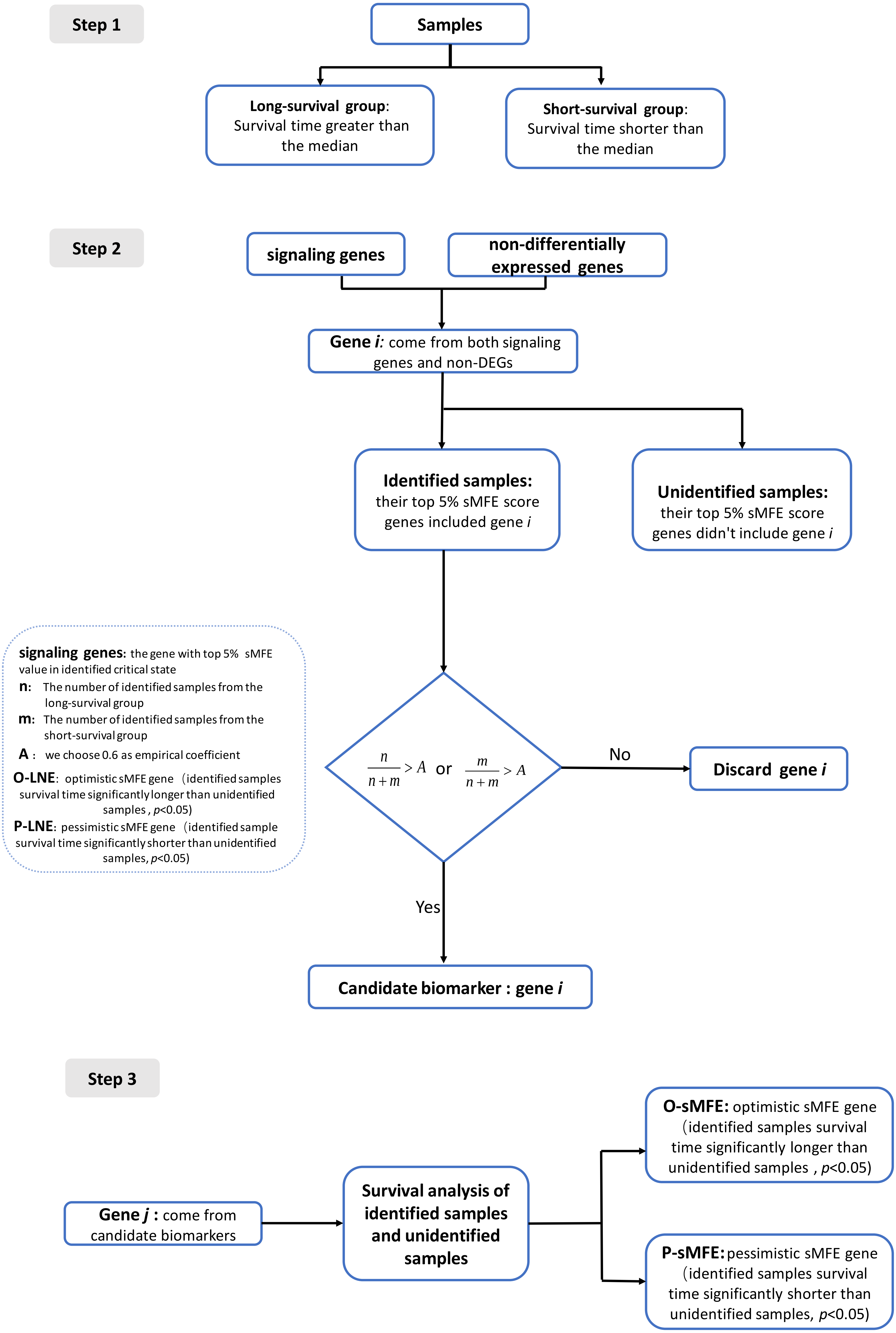

Step 1
Samples
Long-survival group: Survival time greater than the median
Short-survival group: Survival time shorter than the median
Step 2
signaling genes
non-differentially expressed genes
Gene i: come from both signaling genes and non-DEGs
Identified samples: their top 5% sMFE score genes included gene i
Unidentified samples: their top 5% sMFE score genes didn't include gene i
signaling genes: the gene with top 5% sMFE value in identified critical state
n: The number of identified samples from the long-survival group
m: The number of identified samples from the short-survival group
A : we choose 0.6 as empirical coefficient
O-LNE: optimistic sMFE gene (identified samples survival time significantly longer than unidentified samples , p<0.05)
P-LNE: pessimistic sMFE gene (identified sample survival time significantly shorter than unidentified samples, p<0.05)
$\frac{n}{n+m} > A$ or $\frac{m}{n+m} > A$
No
Discard gene i
Yes
Candidate biomarker : gene i
Step 3
Gene j : come from candidate biomarkers
Survival analysis of identified samples and unidentified samples
O-sMFE: optimistic sMFE gene (identified samples survival time significantly longer than unidentified samples , p<0.05)
P-sMFE: pessimistic sMFE gene (identified samples survival time significantly shorter than unidentified samples, p<0.05)
