# Supplementary material for: Identifying the critical state of cancers by single-sample Markov flow entropy
Source: PeerJ. 2023 Jul 24;11:e15695. doi: 10.7717/peerj.15695 (PMC10373650; doi:10.7717/peerj.15695)
Supplement: Supplemental Information 2 — Results of the dynamic network changes for the four cancers. [file peerj-11-15695-s002.pdf]

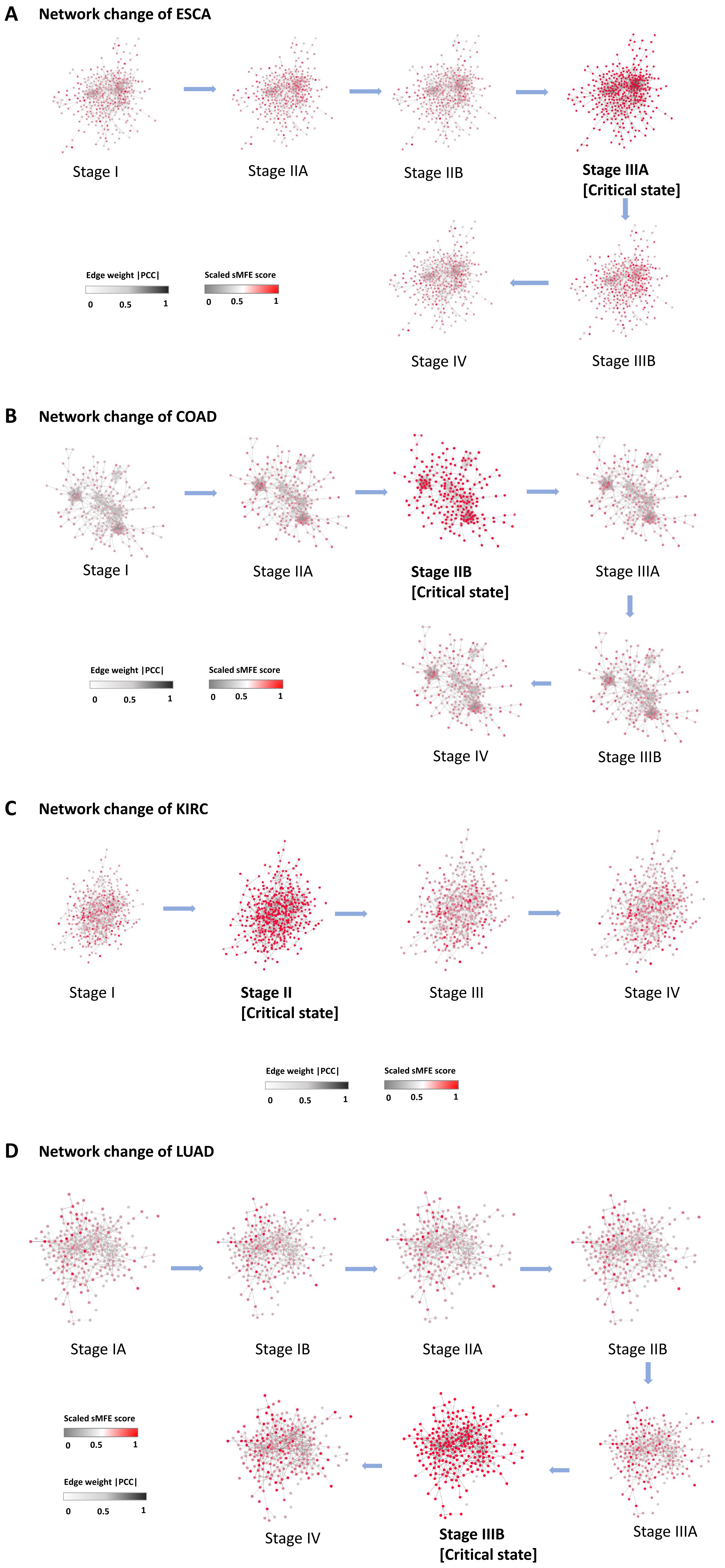
A
Network change of ESCA
Stage I
Stage IIA
Stage IIB
Stage IIIA
[Critical state]
Stage IIIB
Stage IV
Edge weight |PCC|
0
0.5
1
Scaled sMFE score
0
0.5
1
B
Network change of COAD
Stage I
Stage IIA
Stage IIB
[Critical state]
Stage IIIA
Stage IIIB
Stage IV
Edge weight |PCC|
0
0.5
1
Scaled sMFE score
0
0.5
1
C
Network change of KIRC
Stage I
Stage II
[Critical state]
Stage III
Stage IV
Edge weight |PCC|
0
0.5
1
Scaled sMFE score
0
0.5
1
D
Network change of LUAD
Stage IA
Stage IB
Stage IIA
Stage IIB
Stage IIIA
Stage IIIB
[Critical state]
Stage IV
Scaled sMFE score
0
0.5
1
Edge weight |PCC|
0
0.5
1
