# Supplementary figures and images for: Identifying the critical state of cancers by single-sample Markov flow entropy
Source: PeerJ. 2023 Jul 24;11:e15695. doi: 10.7717/peerj.15695 (PMC10373650; doi:10.7717/peerj.15695)

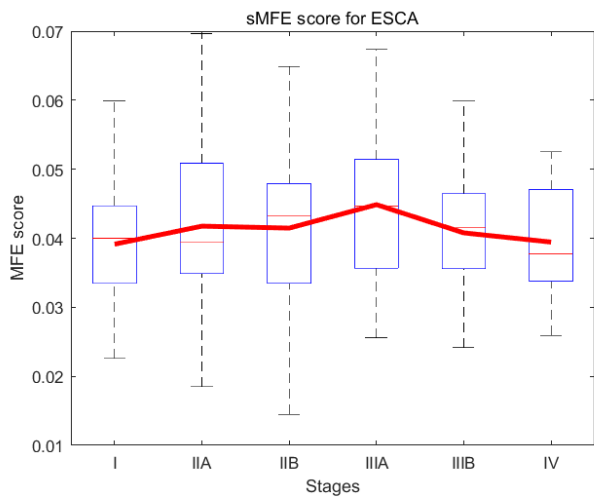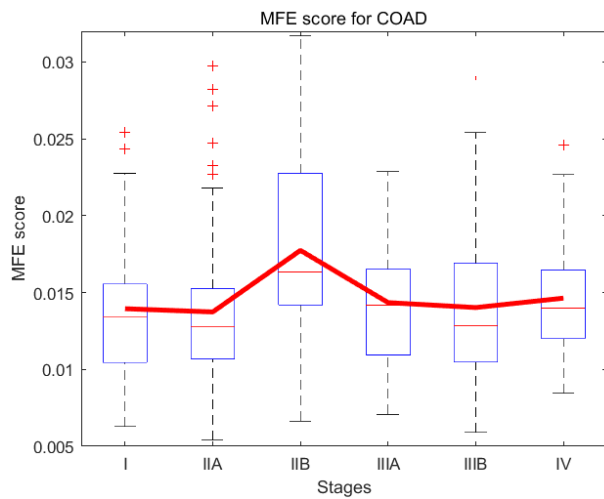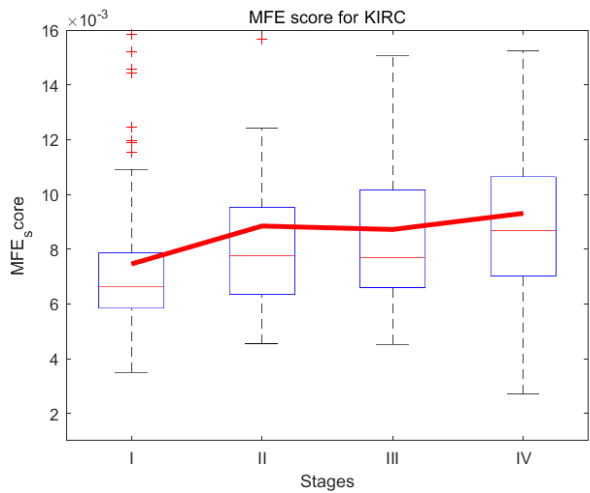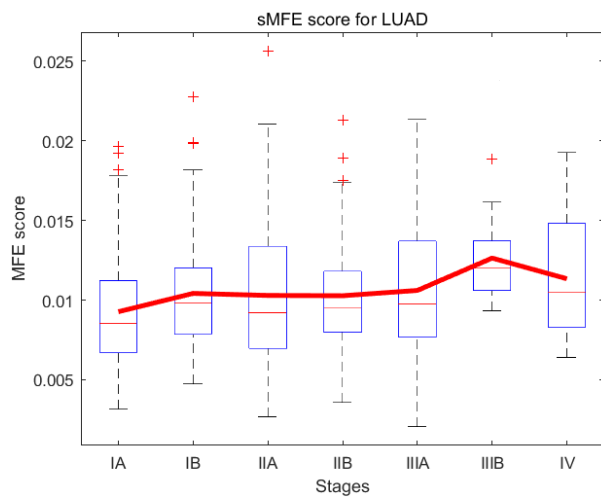

Supplement: Supplemental Information 3 [file peerj-11-15695-s003.pdf]

sMFE score for LIHC

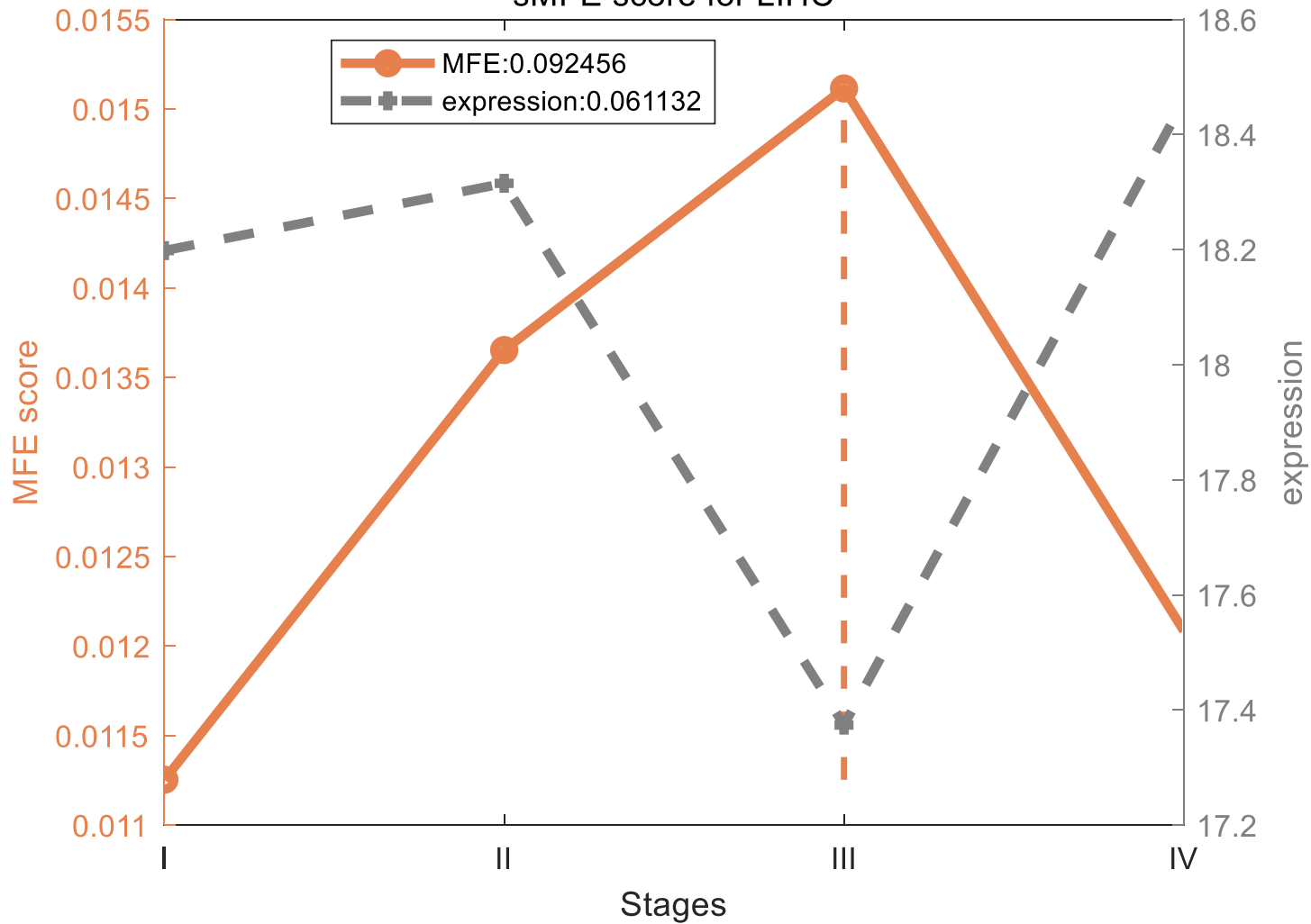

Supplement: Supplemental Information 4 — Based on sMFE score, there is a critical transition around stage III. [file peerj-11-15695-s004.pdf]
