# Supplementary material for: Identifying the critical state of cancers by single-sample Markov flow entropy
Source: PeerJ. 2023 Jul 24;11:e15695. doi: 10.7717/peerj.15695 (PMC10373650; doi:10.7717/peerj.15695)
Supplement: Supplemental Information 5 — TA samples, tumor-adjacent samples. [file peerj-11-15695-s005.docx]

Table S1. The number of tumor samples within each stage in the cancer dataset from TCGA.

|  | TA samples | Stage I | | Stage II | | Stage III | | Stage IV |
| --- | --- | --- | --- | --- | --- | --- | --- | --- |
|  |  | Stage IA | Stage IB | Stage IIA | Stage IIB | Stage IIIA | Stage IIIB |  |
| LUAD | 58 | 106 | 124 | 39 | 59 | 62 | 10 | 21 |
| COAD | 41 | 80 | | 173 | 13 | 28 | 104 | 66 |
| ESCA | 11 | 16 | | 40 | 29 | 35 | 14 | 8 |
| KIRC | 73 | 197 | | 41 | | 112 | | 68 |

TA samples: tumor-adjacent samples
