# Supplementary material for: Identifying the critical state of cancers by single-sample Markov flow entropy
Source: PeerJ. 2023 Jul 24;11:e15695. doi: 10.7717/peerj.15695 (PMC10373650; doi:10.7717/peerj.15695)
Supplement: Supplemental Information 6 [file peerj-11-15695-s006.docx]

Table S2: The optimistic sMFE (O-sMFE) biomarkers and pessimistic sMFE (P- sMFE) biomarkers in ESCA, COAD, KIRC, and LUAD.

| ESCA | | COAD | | KIRC | | | LUAD | |
| --- | --- | --- | --- | --- | --- | --- | --- | --- |
| O-sMFE | **P-sMFE** | **O-sMFE** | **P-sMFE** | **O-sMFE** | **P-sMFE** | | **O-sMFE** | **P-sMFE** |
| SCYL3  TRPS1  USP9Y  CARD10 | EPCAM  PERP  POLDIP3  POLK  SETDB2  UXT | AGR2  LRIG1 PCM1 | ATP1A3 CD9 CRYBA4  CTH  GATA6  MARK1  PARL  PYGL  TNKS1BP1  TOLLIP  ZNF513 | MAML2 MGAM MYSM1  NFRKB  POFUT1  RNF5  TRAF6 | | BST1 CDKN2D  DGCR8  MDGA1  METTL3  MRPL18  RFX5  STK36  VPS39 | ACOX2  KHDRBS3 | ANKRD27 ANP32A FLNC  FZD1  PGM2  SH3RF1 |

Red color: *p*-value < 0.01

Other color: 0.01 < *p*-value <0.05
