# Supplementary material for: Identifying the critical state of cancers by single-sample Markov flow entropy
Source: PeerJ. 2023 Jul 24;11:e15695. doi: 10.7717/peerj.15695 (PMC10373650; doi:10.7717/peerj.15695)
Supplement: Supplemental Information 7 [file peerj-11-15695-s007.docx]

Table S3. How to choose adjustable parameter in GNIPLR.

|  | degree | lambda | P_val |
| --- | --- | --- | --- |
| LUAD | 6 | 0.04 | 0.01 |
| COAD | 6 | 0.012 |  |
| ESCA | 7 | 0.01 |  |
| KIRC | 26 | 0.13 |  |

Tumor-adjacent samples were used to infer the gene regulatory network
